# Transition in vaginal *Lactobacillus* species during pregnancy and prediction of preterm birth in Korean women

**DOI:** 10.1038/s41598-022-26058-5

**Published:** 2022-12-24

**Authors:** Young-Ah You, Sunwha Park, Kwangmin Kim, Eun Jin Kwon, Young Min Hur, Soo Min Kim, Gain Lee, AbuZar Ansari, Junhyung Park, Young Ju Kim

**Affiliations:** 1 Department of Obstetrics and Gynecology, Ewha Medical Research Institute, Ewha Womans University Medical School, Seoul, 07985 Republic of Korea; 2 Department of Obstetrics and Gynecology, Ewha Womans University Mok Dong Hospital, Seoul, 158-051 South Korea; 3 3BIGS CO., LTD., Seoul, Republic of Korea; 4 Graduate Program in System Health Science and Engineering, Ewha Womans University, Seoul, Republic of Korea

**Keywords:** Biomarkers, Microbiology

## Abstract

The predominance of vaginal *Lactobacillus* species, specifically *L. crispatus*, is important for pregnancy maintenance, but varies by race. The composition of the vaginal microbiome can affect susceptibility to adverse pregnancy outcomes. We performed 16S rRNA gene amplicon sequencing on vaginal swabs taken from Korean pregnant women. Here, we report the transition of *Lactobacillus* spp. in samples of full-term birth (FTB) collected longitudinally in the second and third trimesters of pregnancy in a cohort study (n = 23) and their association with *Lactobacillus* abundance and preterm birth (PTB) in a case–control study (n = 200). *Lactobacillus* species, which was dominant in FTB samples including those that received interventions in the second trimester, did not change until 37 weeks of gestation. However, *L. crispatus* was replaced by other *Lactobacillus* species after 37 weeks. The PTB risk showed a closer association with the *Lactobacillus* abundance than with community state type determined by *Lactobacillus* species. PTB was associated with less than 90% of *Lactobacillus* abundance and an increase in *Ureplasma parvum* in the second trimester. Thus, the vaginal microbiome may change in preparation for childbirth in response to multiple intrinsic factors after 37 weeks of gestation. Monitoring the *Lactobacillus* abundance may help improve the reliability of microbial PTB biomarkers.

## Introduction

Preterm birth (PTB) is defined as delivery at less than 37 weeks of gestation and accounts for 8% of childbirths in South Korea^1^. PTB is a leading cause of neonatal and pediatric mortality^2,3^ and is associated with a high susceptibility to various diseases and developmental conditions, such as neurodevelopmental function impairment, cerebral palsy, learning impairment, and visual disorders, which affect the long-term physical health of the offspring^4,5^. Spontaneous preterm labor and preterm premature rupture of fetal membranes (PPROM) have been reported in 75% of PTBs^6^. The remaining 25% of PTBs are associated with maternal or fetal conditions, such as preeclampsia or intrauterine growth restriction (IUGR)^7^. Labor is caused by three physiological processes: dilatation of the cervix, contraction of the uterus, and rupture of the amniotic membrane. In PTB, these phenomena occur in response to pathological processes. A better understanding of the etiology of PTB is necessary to improve patient stratification for targeted therapeutic interventions and develop novel therapeutic strategies.

Pregnancy is accompanied by a shift in the structure of the vaginal bacterial community, with the typical predominance of one or two *Lactobacillus* species^8–11^. These bacteria produce metabolites, such as lactic acid, to lower the vaginal pH, and secrete antibacterial bacteriocins, which can alter their tolerance to the anaerobic microbial community^12^. Dysbiosis of the vaginal microbiome is associated with an increased risk of adverse pregnancy outcomes, especially PTB, which increases the levels of certain cytokines, such as interleukin (IL) 6 and IL-8^13–15^. Furthermore, the maternal vaginal microbiome may also serve as an important source of the neonatal gut microbiome^16,17^, which exerts a profound effect on host metabolism and immunity^18,19^.

The vaginal microbiome is important for reproductive tract health and maintenance of pregnancy. The community state type (CST) is a classification system based on the predominant *Lactobacillus* species present or a *Lactobacillus*-depleted status: CST I, *L. crispatus*; CST II, *L. gasseri*; CST III, *L. iners*; CST V, *L. jensenii*; CST IV, *Lactobacillus-*depleted group^20^. In general, because the dominance of *L. crispatus* suppresses pathogen colonization, it is often reported to protect against early onset neonatal sepsis associated with PPROM and cervical shortening and lower the risk of PTB^20–23^. Conversely, a *Lactobacillus*-depleted status (CST IV) is usually accompanied by a significant increase in the abundance of *Gardnerella vaginalis*, *Prevotella* species, *Atopobium vaginae*, *Sneathia* species, and other bacterial vaginosis-associated bacteria, primarily owing to the depletion of typical vaginal microbiota^24,25^. This community state is associated with an increased risk of PTB. However, the taxa related to a higher PTB risk reported in different studies are not consistent and possibly differ based on the ethnicity and area of residence of the recruited study participants^20–22^.

In our previous study, we reported that in Korean women, the association of the vaginal microbiome with PTB was more strongly indicated in *Lactobacillus* abundance-based classification than in CST-based classification^26^. We then reported the bacterial risk score for PTB prediction based on the ratio of *L. iners* and *Ureaplasma parvum* abundances^27^. Recently, we reported that *Ureaplasma* and *Prevotella* abundances, along with *Lactobacillus* abundance, are associated with full-term birth (FTB)^28^. Here, we analyzed the characteristics of the vaginal microbial community during pregnancy in a cohort of Korean women and determined whether the characteristics of a particular community were associated with the risk of PTB in a case–control study.

## Results

### Transition of vaginal *Lactobacillus* species during pregnancy and in the postpartum period in longitudinal samples

Metataxonomic profiling of vaginal bacteria was performed using 55 swabs collected from 23 women with FTB in a cohort study. At the species level, the following CSTs were identified: CST I, *L. crispatus* (52%); CST II, *L. gasseri* (9%); CST III, *L. iners* (26%); CST V, *L. jensenii* (0%); and CST IV (13%) in the second trimester (n = 23), and CST I, *L. crispatus* (52%); CST II, *L. gasseri* (9%); CST III, *L. iners* (13%); CST V, *L. jensenii* (9%); and CST IV (17%) in the third trimester (n = 23). Most of the CST in 6 weeks postpartum were CST IV (n = 9).

The frequency of each CST did not appear to change between the second and third trimester. However, among 4 out of 12 samples with CST I in the second trimester, one sample showed transition to CST III, two samples showed transition to CST IV, and one sample showed two dominant *Lactobacillus* species (CST I and CST V), in the third trimester. Interestingly, all samples were collected at more than 37 weeks of gestation (Table 1). Among pregnant women treated with antibiotics, antifungal antimicrobials, or vaginal progesterone after the collection of vaginal fluid in the second trimester, 60% showed no change in CSTs in the third trimester. The remaining 40% of the samples showed *Lactobacillus* transition and these samples were collected at more than 37 weeks of gestation (Table 2).

**Table 1:**
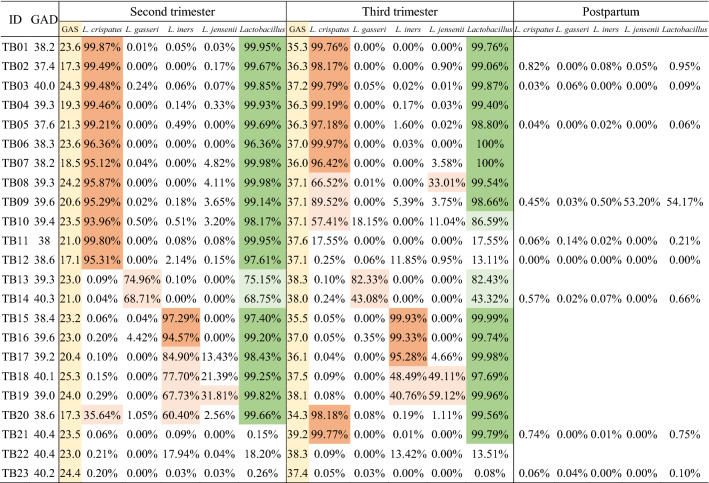
The predominant *Lactobacillus* species in each trimester during pregnancy in women with term delivery (n = 23).

**Table 2:** Characteristics of the participants in the cohort study.

ID	GAD	Age	BMI	CL (mm)	WBC (10^6^/mL)	Antibiotics/Progesterone therapy	Mode of delivery	Reason for delivery
TB01	38.2	38	21.1	–	10.1	Antifungal antimicrobials	ND	Full-term labor
TB02	37.4	29	20.7	30.3	12.3	Antifungal antimicrobials, Antibiotics	C/S	Placental abruption
TB03	40.0	28	18.8	23.0	8.3	Progesterone VT	ND	Full-term labor
TB04	39.3	34	21.2	25.2	10.0	Progesterone VT	ND	Full-term labor
TB05	37.6	37	20.6	31.6	8.9	Antibiotics	C/S	Full-term labor
TB06	38.2	28	20.4	31.0	10.1	Antibiotics, Progesterone VT	C/S	Placental abruption
TB07	39.6	35	20.5	–	–	Antifungal antimicrobials	ND	Full-term labor
TB08	39.3	33	18.6	–	5.7	Antifungal antimicrobials, Antibiotics	ND	Full-term labor
TB09	39.4	29	21.2	–	–	Antifungal antimicrobials	C/S	Full-term labor
TB10	38.3	30	21.8	40.0	7.5	Antifungal antimicrobials	ND	PROM
TB11	38.0	44	24.2	30.0	8.9	None	ND	PROM
TB12	38.6	21	24.0	28.2	10.5	Antibiotics	ND	PROM
TB13	39.3	33	22.9	–	13.3	Antifungal antimicrobials	ND	PROM
TB14	40.3	35	21.8	24.3	11.2	Antibiotics	ND	Full-term labor
TB15	38.4	34	21.5	30.8	8.0	None	ND	Full-term labor
TB16	39.6	37	19.0	30.0	8.7	None	ND	Full-term labor
TB17	39.2	28	21.1	29.7	9.2	Antifungal antimicrobials	ND	Full-term labor
TB18	40.1	31	17.3	27.0	7.1	Antifungal antimicrobials	ND	Full-term labor
TB19	39.0	30	25.0	35.0	10.2	Progesterone VT	C/S	Full-term labor
TB20	38.6	39	21.1	27.7	4.7	Antibiotics, Progesterone VT	ND	Full-term labor
TB21	40.4	32	22.0	39.0	8.6	Antibiotics	ND	PROM
TB22	40.4	29	19.8	23.4	8.1	Progesterone VT	ND	Full-term labor
TB23	40.2	33	24.6	26.5	8.5	Antifungal antimicrobials, Progesterone VT	ND	Full-term labor

*BMI* Body-mass index, *CL* Cervical length, *VT* Vaginal tablet, *PROM* Premature rupture of membr.

### Community profiling of the vaginal microbiome in pregnant women with PTB

In this case–control study, 200 Korean women (126 with FTB and 74 with PTB) were included. Vaginal fluid samples were collected between 14 weeks of gestation and 6 weeks postpartum. We investigated whether the vaginal microbiome composition at the time of sampling was related to pregnancy maintenance or delivery including PTB. Based on the gestational age at sampling, we categorized the patients into five groups: 56 at time point A (14–23 weeks), 43 at time point B (24–31 weeks), 54 at time point C (32–36 weeks), 41 at time point D (more than 37 weeks), and 36 at time point E (6 weeks postpartum). The samples were targeted at time points A to C for comparison between FTB and PTB, since women whose samples were collected after more than 37 weeks were considered a part of the full-term delivery group. The characteristics of the study participants are listed in Table 3. PTB occurred in 74 women with gestational age < 37 weeks, with 56 women (75.7%) at 32–36 weeks, and 18 women (24.3%) at < 32 weeks. Preterm labor occurred in 28 patients (39.4%), PPROM occurred in 21 patients (29.6%), and other medical indications with preterm labor were observed in 20 patients (28.1%).

**Table 3:** Clinical characteristics of participants in the case–control study (n = 200).

	Term (≥ 37, *n* = 126)	Preterm (< 37, *n* = 74)	*p*-value
	Mean ± SD	Mean ± SD	
Maternal age	32.6 ± 4.0	32.6 ± 5.6	0.988
Pre-pregnancy BMI (kg/m^2^)	20.2 ± 5.3	20.5 ± 6.0	0.691
Cervical length (mm)	29.7 ± 8.7	24.5 ± 10.4	0.003^a^
**Mode of delivery**			0.001*
Vaginal, *n* (%)	87 (64.9)	21 (28.4)	
C-section, *n* (%)	47 (35.1)	53 (71.6)	
Gestational age at sampling (weeks)	30.0 ± 7.3	23.3 ± 5.8	0.458
14–27, *n* (%)	60 (20.6)	24 (31.1)	
28–36, *n* (%)	25 (13.5)	50 (50.0)	
≥ 37 completed	41 (32.5)	0	
Gestational age at delivery (weeks)	39.1 ± 1.0	32.8 ± 3.8	< 0.001^a^
< 32 completed, *n* (%)	0	18 (24.3)	
32–36 completed, *n* (%)	0	56 (75.7)	
≥ 37 completed, *n* (%)	126 (100)	0	
White blood cell, (× 10^3^ cells/μL)	9.0 ± 2.0	10.8 ± 3.4	< 0.001^a^
**Clinical characteristics**			
Preterm labor, *n* (%)		28 (39.4)	
Preterm PROM, *n* (%)		21 (29.6)	
Medical indication^§^, *n* (%)		20 (28.1)	
Missing, *n* (%)		2 ( 2.8)	
Birth weight (g)	3261.4 ± 310.7	2012.7 ± 719.5	< 0.001^a^
**Sex**			0.661
Male, *n* (%)	79 (59.0)	46 (62.2)	
Female, *n* (%)	55 (41.0)	28 (37.8)	
Apgar 1 min	9.4 ± 1.3	6.9 ± 2.8	< 0.001^a^
Apgar 5 min	9.8 ± 0.7	8.3 ± 2.1	< 0.001^a^

*BMI* Body mass index.

^§^Pregnant women with medical indication were diagnosed with preterm labor.

^a,^ Student’s t-test.

* χ2 test. Data are shown as the mean ± SD for continuous variables and as n (%) for categorical data.

**Figure 1:**
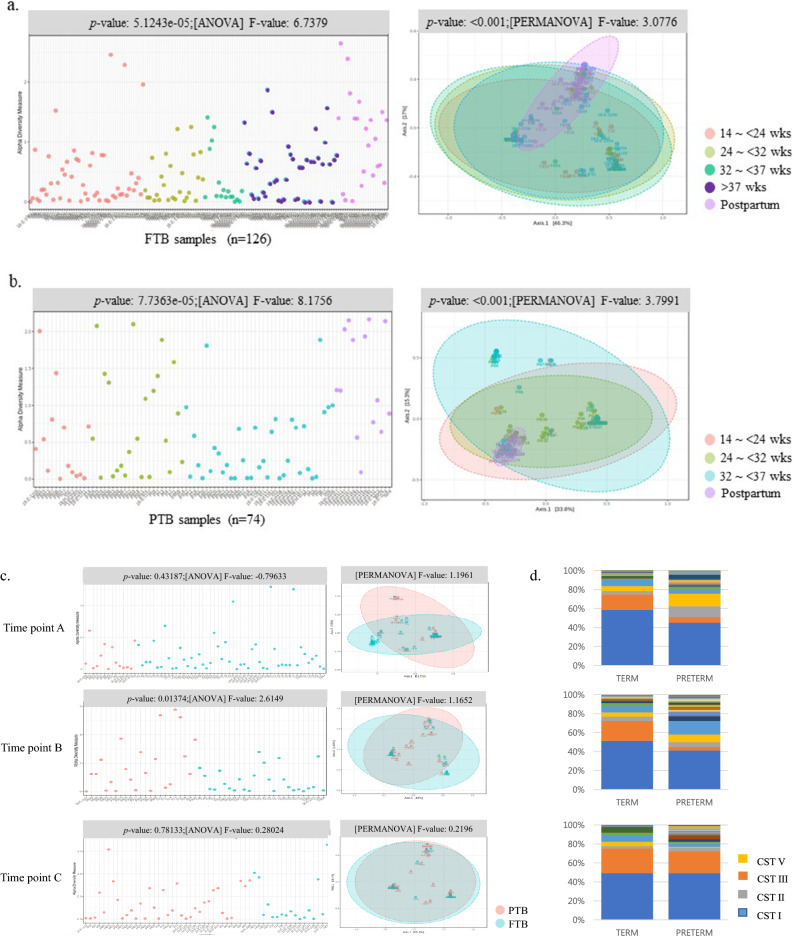
The alpha and beta diversities of the vaginal microbiome. The alpha diversity of the vaginal microbiome at each time point of sampling in women with (**a**) term delivery and (**b**) preterm delivery and (**c**) between FTB and PTB samples at each time point. (**d**) Bar graph of the vaginal microbiome at each time point. Based on the gestational age at sampling, we divided the samples into five groups: 56 at time point A (14–23 weeks), 43 at time point B (24–31 weeks), 54 at time point C (32–36 weeks). *FTB* Full-term birth, *PTB* Preterm birth, *PP* postpartum.

The Shannon diversities of FTB or PTB samples differed significantly between the indicated time points (Fig. 1a,b). The alpha diversity between FTB and PTB samples differed significantly only at sampling time point B (Fig. 1c). Following this, the beta diversity was calculated between the groups using the Bray–Curtis dissimilarity index for samples collected from each participant, and the indices were used to create a principal coordinates analysis (PCoA) ordination plot. The beta diversities of FTB or PTB samples differed significantly between the indicated time points, but the beta diversities between FTB and PTB samples at each sampling time point did not differ significantly. In addition, the CST and *Lactobacillus* abundance did not differ significantly (Fig. 1d).

At the species level, six CSTs were identified, among which four were dominated by a single species of *Lactobacillus* (CST I (48.5%), CST II (5.5%), CST III (16.5%), and CST V (4.0%)), one was dominated by two *Lactobacillus* spp. (6.0%), and one was *Lactobacillus*-depleted CST IV (19.5%, Table 4). No significant difference was observed between the frequencies of the CSTs in the FTB and PTB samples or based on classification by gestational age at sampling (Supplementary Table 1). The frequencies of CSTs did not differ significantly between indications for delivery (Supplementary Table 2).

**Table 4:** Analysis based on CST groups (I, II, III, and V) representing the dominant *Lactobacillus* species in women sampled at different weeks of gestation (n = 159).

CST	Dominant species	Total		Term		Preterm		*p*-value
		*n*,	%	*n*,	%	*n*,	%	
I	*L. crispatus*	84	52.8	48	56.5	36	48.6	0.359
II	*L. gasseri*	8	5.0	3	3.5	5	6.8	
III	*L. iners*	29	18.2	18	21.2	11	14.9	
IV	Depletion	30	18.9	12	14.1	18	24.3	
V	*L. jensenii*	8	5.0	4	4.7	4	5.4	
	Total	159		126		74		

Statistical analysis was performed by Fisher’s exact test. *CST* Community state type.

Based on the relative bacterial abundances at the genus level, samples were grouped as either *Lactobacillus*-dominant (> 90%, 61% of samples) or *Lactobacillus*-depleted (≤ 90%, 39% of samples). Based on this classification, the frequencies in the full-term and preterm groups were determined (Supplementary Table 3). Participants in the *Lactobacillus*-depleted group showed a greater frequency of PTB than participants in the *Lactobacillus*-dominant group (*p* < 0.05). Based on classification by gestational age at sampling, in both *Lactobacillus*-dominant and *Lactobacillus*-depleted groups, the frequencies of FTB and PTB determined at time points A and B were significantly different (*p* < 0.05), whereas those determined at time point C were not (Table 5). Significant differences were observed between indications for delivery (Supplementary Table 4).

**Table 5:** Comparison between the frequencies of FTB and PTB according to vaginal *Lactobacillus* abundances (n = 159).

Sampling time point	Percentile	Total	Term		Preterm		*p*-value
		*n,*	*n,*	%	*n,*	%	
14–23 weeks	> 90%	37	32	(76.2)	5	(35.7)	0.009
	≤ 90%	19	10	(23.8)	9	(64.3)	
24–31 weeks	> 90%	25	18	(69.2)	7	(30.4)	0.010
	≤ 90%	24	8	(30.8)	16	(69.6)	
32–36 weeks	> 90%	38	13	(76.5)	25	(67.6)	0.749
	≤ 90%	16	4	(23.5)	12	(32.4)	

Statistical analysis was performed by Fisher’s exact test.

*FTB* Full-term birth, *PTB* Preterm birth.

### Comparative analysis of the vaginal microbiome in FTB and PTB

**Figure 2:**
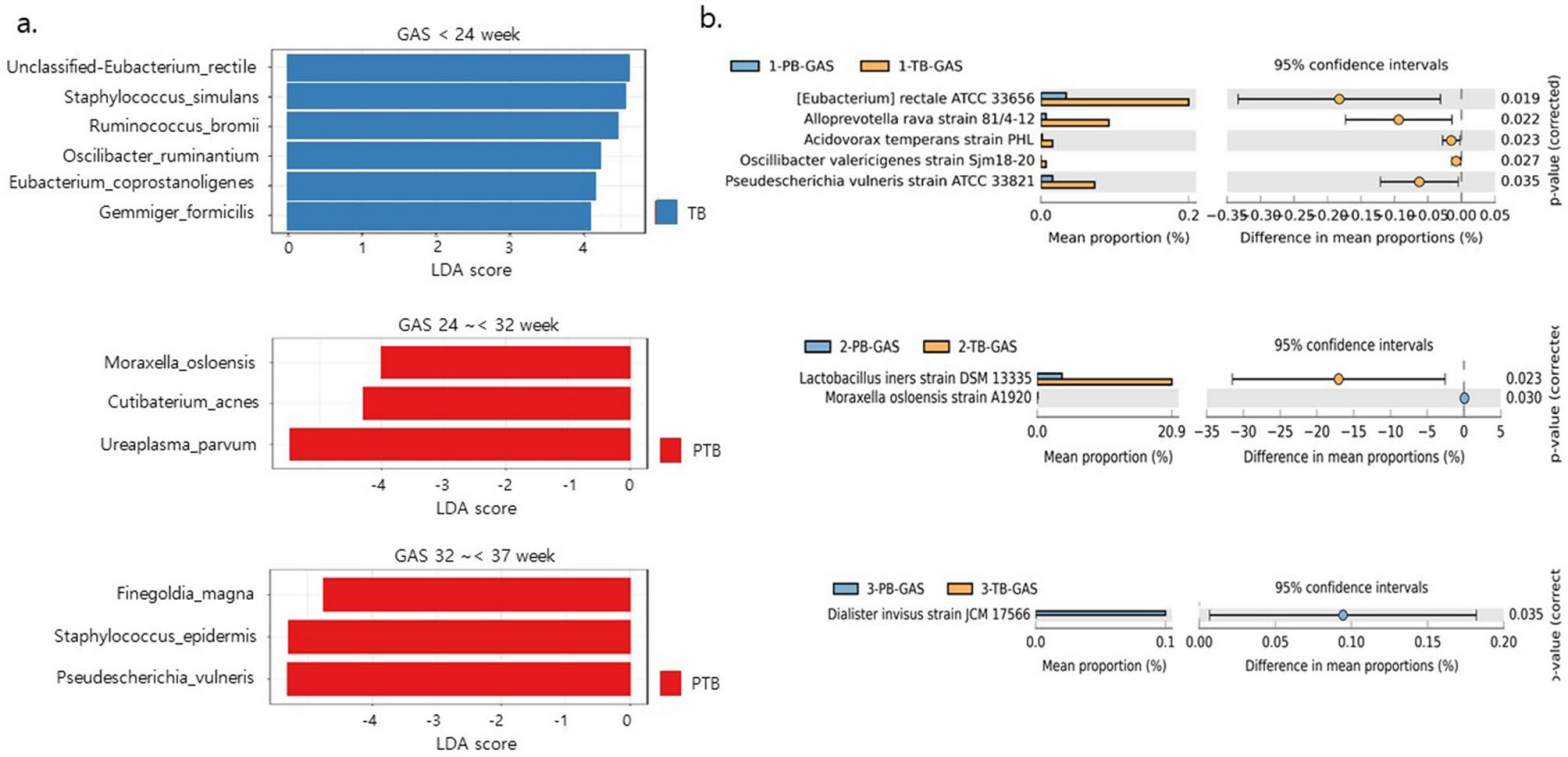
Comparative analysis of the vaginal microbiome in FTB and PTB. (**a**) FTB- or PTB-associated microbiome using linear discriminant analysis effect size, (**b**) differential abundance of the vaginal microbiome in women with FTB and PTB at each sampling time point. *FTB* Full-term birth, *PTB* Preterm birth.

We performed linear discriminant analysis effect size (LEfSe) at each sampling time point to identify the biomarkers of PTB. At time point A, six microbiomes that contained *Ruminococcus bromii* and *Gemmiger formicilis* were significantly associated with FTB (*p* < 0.05). Three microbiomes that contained *U. parvum* at time point B and three microbiomes that contained *Staphylococcus epidermis* were significantly associated with PTB (*p* < 0.05). However, we did not identify any microbiome that was associated with PTB at all three time points (Fig. 2a).

We analyzed the differential abundance of microbes at the taxonomic level at each time point using STAMP^29^ (Fig. 2b). In this analysis, some microbiota analyzed using LEfSe were also detected in PTB samples. *L. iners*, which was not detected using LEfSe, was found to be significantly associated with FTB at time point B (*p* < 0.05).

### Prediction of PTB

Logistic regression analysis was performed to predict PTB (Table 6). We considered the abundances of *L. iners* and *U. parvum* based on the PTB-associated microbiota reported in our previous study. A logistic regression mixed-effects model indicated an association between PTB and the *Lactobacillus*-depleted state (≤ 90%) (*p* < 0.001), sampling time point (*p* < 0.001), and *U. parvum* abundance (*p* = 0.04).

**Table 6:** Logistic regression analysis for predicting preterm birth.

	beta	*p*-value	Exp (B)	95% CI	
				Lower	Upper
Intercept	−4.25	< 0.001	0.014		
*Lactobacillus*-depleted state	1.24	0.001	3.456	1.627	7.343
Sampling time point	1.08	< 0.001	2.821	1.795	4.433
*Lactobacillus iners* abundance	0.15	0.696	1.158	0.554	2.421
*Ureaplasma parvum* abundance	0.49	0.177	1.636	0.800	3.344

beta-values estimated using the multivariable logistic regression model.

## Discussion

We compared the vaginal microbiome composition during pregnancy in a cohort study and the abundance of the vaginal microbiome in women with FTB and PTB in a case–control study. Treatment with antibiotics or adjuvant vaginal progesterone in the second trimester might not affect the transition of predominant *Lactobacillus* species in the vaginal microbiome to other *Lactobacillus* species in the third trimester. In the cohort study, after 37 weeks of gestation, the abundance of predominant *Lactobacillus* species decreased, or the species were replaced by other *Lactobacillus* species. In the case–control study, the frequencies of CST did not differ significantly between the term and preterm groups. The *Lactobacillus*-depleted group (≤ 90%) showed a significant occurrence of PTB with a higher abundance of *Ureaplasma parvum*. The findings of this study suggest that the vaginal *Lactobacillus* abundance and the relative abundance of *U. parvum* during pregnancy may be a novel strategy for stratifying pregnant women for the prediction of PTB.

Compared to that in non-pregnant women, the diversity of the vaginal microbiome generally decreases in pregnant women, and the microbiome gradually becomes enriched with *Lactobacillus* species^10^. During pregnancy, the estrogen level, which increases drastically owing to placental production, affects vaginal epithelial cell maturation and glycogen accumulation, thus influencing *Lactobacillus* colonization^30,31^. In our cohort, the vaginal microbiome of most pregnant women was predominated by *Lactobacillus* species, and in women with FTB in the case–control experiment as well as in women from the cohort experiment, a stable signature corresponding to the gestational age was observed before 37 weeks of gestation. These results are consistent with an earlier observation that the stability of the vaginal microbiome tends to increase with gestational age, with an increase in the predominance of *Lactobacillus* species^32^. However, after 37 weeks of gestation, the dominant *Lactobacillus* species, especially *L. crispatus*, were replaced by other species, or a *Lactobacillus-*depleted state was established, in approximately 40% of the cohort participants. Even though previous studies have reported that the dominant *Lactobacillus* species are unlikely to change during pregnancy, this difference could be attributed to the race of the individual and the gestational age at sampling. We suggest that the transition after 37 weeks of gestation could indicate physiological preparation for childbirth.

The dominance of the vaginal microbiome during pregnancy is altered dynamically, and during the postpartum period, any *Lactobacillus* species that is sensitive to estrogen and is dominant in the vaginal microbiome is depleted with the decline in estrogen levels^33–35^. Consistent with this, in both the cohort and case–control studies, the vaginal microbiome of the patients (as detected in samples collected postpartum) had a lower abundance of *Lactobacillus* species. Treatment with antibiotics, antifungal antimicrobials, and vaginal progesterone did not appear to affect the vaginal microbiome during pregnancy. Most vaginal samples from the third trimester were collected at 3–4 weeks after treatment in the second trimester. Consistent with our results, progesterone therapy did not appear to affect the relative abundance of *Lactobacillus* species or the species diversity in vaginal samples^23,36^. This result indicates that during pregnancy, progesterone is not likely to act by modulating the vaginal microbiome. However, Brown et al. reported that in women in whom *Lactobacillus* spp. was dominant before erythromycin treatment, the treatment was associated with a transition toward *Lactobacillus* depletion^22^. This discrepancy may be attributed to the different sample collection time points after antibiotic therapy. Thus, our results support the finding that *Lactobacillus* species present initially may be predominant in the vaginal microbiome for a certain period, even if the composition of the vaginal microbiome changes immediately after antibiotic treatment.

A lower diversity of the vaginal microbiome with the dominance of *Lactobacillus* species is usually associated with a healthy pregnancy^8–11^. Our results showed no significant differences between the alpha diversities in samples collected at different sampling time points from women with FTB and PTB. There were no significant differences in the frequencies of the CST constructs in the vaginal microbiome between FTB and PTB samples. However, in the classification based on *Lactobacillus* abundance, the *Lactobacillus*-dominant (> 90%) group showed significantly higher frequencies of FTB, whereas the frequencies of PTB were significantly higher in the *Lactobacillus*-depleted (≤ 90%) group. The indications for delivery were also significantly different between the *Lactobacillus*-dominant and *Lactobacillus*-depleted groups. These results confirmed our previous findings on *Lactobacillus* abundance-based classification^26^. In contrast, some studies have reported that the perceived benefits of *Lactobacillus* dominance in pregnancy are species-specific; *L. crispatus* abundance has been associated with term delivery, whereas *L. iners* abundance has been associated with an increased risk of preterm delivery^23,37^. However, Romero et al. reported that the composition and abundance of vaginal microbiome did not differ between mothers who delivered preterm and at term in a primarily African-American cohort^10^.

In our previous study, we used machine learning with the ratio of relative abundances of *L. iners* and *U. parvum* for PTB prediction^27^. We also reported that *Ureaplasma* and *Prevotella* colonization with *Lactobacillus* during pregnancy facilitates FTB^28^. In this study, in the logistic regression analysis, we included the *L. iners* and *U. parvum* abundances and the gestational age at sampling as adjusted factors to analyze the risk of PTB. DiGiulio et al. reported that a low abundance of *Lactobacillus* and high abundance of *Gardnerella* or *Ureaplasma* were associated with an increased risk of PTB in a largely Caucasian cohort^38^. Callahan et al. also identified an association between *Gardnerella* abundance and PTB, but only in one cohort primarily comprising Caucasian women^39^. Notably, the ethnic and racial demographics in these studies varied significantly, suggesting that vaginal dysbiosis may differ depending on host factors^40,41^. We found that a decline in the abundance of *Lactobacillus* was associated with PTB. In addition, our results indicate *U. parvum* abundance is an important risk factor for PTB prediction in the *Lactobacillus*-depleted state.

In summary, our results indicate that specific characteristics of the vaginal microbiome, including the *L. crispatus* dominant state, can change during pregnancy, and at the genus level, the *Lactobacillus*-depleted state is associated with PTB. The vaginal microbial composition at any time point of sampling may be used for stratifying the risk of PTB; a *Lactobacillus*-dominant state is predictive of FTB, whereas a *Lactobacillus*-depleted state is associated with an increased risk of PTB. Although the sample size was small, the treatment with antibiotics, antifungal antimicrobials, and vaginal progesterone in women with FTB did not appear to adversely affect the relative abundance of vaginal *Lactobacillus* species or the species diversity during pregnancy. This treatment is not likely to act by modulating vaginal microbial communities. Further studies are needed to investigate the association between changes in the vaginal microbiome after 37 weeks of gestation and the course of labor.

## Methods

### Participants and clinical data

We analyzed changes in the vaginal microbiome in samples of full-term birth (FTB) collected longitudinally in a cohort study (n = 23) and compared the composition of the vaginal microbiome between FTB and PTB samples collected in a case–control study (n = 200). First, we recruited women with singleton pregnancies at 14 ~ 27 + 6 weeks of gestation and collected vaginal swabs longitudinally in the second and third trimesters of gestation and 6 weeks postpartum from the cohort. Second, in the case–control study, we collected samples once from women with singleton pregnancies during pregnancy. The PTB group was classified based on the clinical findings of preterm labor, PPROM, and medical indications with preterm labor. Among the medical indications, placental abruption, IUGR, preeclampsia, and placenta previa were considered. The FTB group included samples collected in the third trimester of the cohort study. The participants were followed-up throughout the prenatal course, with serial vaginal swabs obtained during routine prenatal visits. Demographic data, medical history, and clinical obstetric outcome data were collected by obstetricians and recorded in the electronic medical record system.

The study was conducted in accordance with the guidelines of the Declaration of Helsinki and was approved by the Institutional Review Board of Ewha Womans University Medical Center (EUMC 2018-07-007-010). Participants provided written informed consent to take part in the study.

### Sample collection and DNA extraction

Vaginal samples were collected from the posterior fornix and applied to the lateral walls of the vaginal canal (three to five times on each sidewall). The swab was then placed into a sterile collection tube, immediately stored at − 20 °C until transportation to the laboratory, and frozen at − 80 °C until DNA extraction. Genomic DNA (gDNA) was extracted from the sample using a DNeasy PowerSoil Kit (Qiagen) in accordance with the manufacturer’s instructions. The concentration of the extracted gDNA was measured using a UV spectrophotometer.

### 16S rRNA gene sequencing and sequence data processing

The V3 and V4 hypervariable regions of the 16S rRNA gene were amplified by PCR using barcoded universal primers (Supplementary Table 5). Sequencing was performed using the Illumina MiSeq platform (Illumina, Inc. San Diego, CA, USA), according to the manufacturer’s instructions. Raw sequence data were analyzed using the QIIME2 (v.2020.11) bioinformatics pipeline. The 300 bp paired-end reads obtained from the Illumina MiSeq platform for each sample were demultiplexed to attribute sequence reads to the appropriate samples and joined. The sequence reads were denoised and dereplicated into amplicon sequence variants (ASVs) using the DADA2 tool, which also filters chimeras. Each read sequence was trimmed to 388 bp. A total of 20,220,280 sequences of the 16S rRNA gene and 7,100 features were generated from 268 swab samples, with a mean frequency of 75,448 sequences per sample. A feature table, equivalent to the ASV table generated using QIIME2, was generated for all samples with a mean frequency of 2,847. The feature table was used for taxonomic classification, alpha and beta diversity analyses, and differential abundance measurements in the different experimental groups. Taxonomy was assigned to each ASV using the SILVA (version 138) database and a fitted classifier classification-sklearn method. Species-level assignments were performed using the BLASTn software (https://blast.ncbi.nlm.nih.gov/). The highest percentage of identity and expectation values were considered when selecting significant BLAST hits.

### Statistical analysis

The clinical characteristics and outcomes of pregnant women who delivered term vs. preterm were analyzed using a Student’s *t*-test for continuous variables and Fisher’s exact test for categorical variables. We performed logistic regression analysis to predict PTB. Statistical analyses were conducted using SPSS software ver. 21.0 (IBM). All analyses were two-tailed, and *p* < 0.05 was considered to indicate statistical significance.

To explore the alpha diversity of the vaginal microbiome, we calculated the Shannon diversity index for each host and used the indices to prepare a sample-level dot plot and group-level box plot. To explore the beta diversity of the vaginal microbiome, we calculated the Bray–Curtis dissimilarity index for each host and used the indices to create a non-metric multidimensional scaling (NMDS) and PCoA ordination plot.

To analyze the vaginal microbiome in the different groups, we used the non-parametric Kruskal–Wallis rank sum test to identify features with significant differential occurrences in different groups, followed by LEfSe to evaluate the effect size of the significant features. Multivariate analyses, such as principal coordinates analysis and NMDS, were performed. The adjusted p-value was calculated by adjusting the false-positive rate using the false-discovery rate (FDR). Correlations between the taxa and sample groups were analyzed using the Pearson’s correlation coefficient r as the distance measure. Statistical analyses were performed using R software (version 3.6.2), and microbiome analysis was performed using MicrobiomeAnalyst (https://www.microbiomeanalyst.ca/)^42,43^. Differential abundances at each taxonomic level were tested using STAMP, with two-sided Whites non-parametric *t*-tests and Benjamini–Hochberg FDR correction for multiple testing.

The taxonomic profiles of vaginal microbial communities were sorted into CSTs, which were discrete categories. The term “CST” is used in microbial ecology to describe a group of community states with similar microbial phylotype compositions and abundances. This type of grouping is useful for reducing dimensionality. This approach is advantageous because collapsing a hyper-dimensional taxonomic profile into a single categorical variable facilitates processes such as data exploration, epidemiological studies, and statistical modeling.

## Supplementary Information

Supplementary Information.

## Data Availability

The datasets generated and/or analyzed during the current study are available in the Sequence Read Archive repository, SUB11962593.
