# Supplementary material for: Transition in vaginal Lactobacillus species during pregnancy and prediction of preterm birth in Korean women
Source: Sci Rep. 2022 Dec 24;12:22303. doi: 10.1038/s41598-022-26058-5 (PMC9789976; doi:10.1038/s41598-022-26058-5)
Supplement: Supplementary file 1 — Supplementary Information. [file 41598_2022_26058_MOESM1_ESM.docx]

**Transition in vaginal *Lactobacillus* species during pregnancy and prediction of preterm birth in Korean women**

Young-Ah You^1^, Sunwha Park^1^, Kwangmin Kim^3^, Eun Jin Kwon^1^, Young Min Hur^1^, Soo Min Kim^1,2^, Gain Lee^1,2^, AbuZar Ansari^1^, Junhyung Park^3^, Young Ju Kim^1,2^*

^1^Department of Obstetrics and Gynecology and Ewha Medical Research Institute, Ewha Womans University Medical School, Seoul 07985, Republic of Korea

^2^Graduate Program in System Health Science and Engineering, Ewha Womans University, Seoul, Republic of Korea

^3^ 3BIGS CO., LTD., Seoul, Republic of Korea

Running Head:  Transition in vaginal *Lactobacillus* and prediction of PTB

The authors declare no conflict of interest.

***Corresponding authors: Young Ju, Kim**

Department of Obstetrics and Gynecology, Ewha Womans University Mok Dong Hospital, Seoul 158-051, South Korea; E-mail: kkyj@ewha.ac.kr; Tel: +82-2-2650-5029; Fax: +82-2-2647-9860

**Supplementary Information**

**Supplementary Table 1.** Analysis of the CST groups (I, II, III, and V) representing the dominant *Lactobacillus* species in women sampled at each time point (n=200)

| CST | Dominant  species | Total | | 14~23 weeks of gestation | | | | 24~31 weeks of gestation | | | | 32~36 weeks of gestation | | | | more than 37 weeks | |
| --- | --- | --- | --- | --- | --- | --- | --- | --- | --- | --- | --- | --- | --- | --- | --- | --- | --- |
|  |  |  |  | Term | | Preterm | | Term | | Preterm | | Term | | Preterm | | Term | |
|  |  | *n,* | % | *n,* | % | *n,* | % | *n,* | % | *n,* | % | *n,* | % | *n,* | % | *n,* | % |
| I | *L. crispatus* | 97 | 48.5 | 25 | 59.5 | 7 | 50.0 | 14 | 53.8 | 11 | 47.8 | 9 | 52.9 | 18 | 48.6 | 17 | 41.5 |
| II | *L. gasseri* | 11 | 5.5 | 2 | 4.8 | 2 | 14.3 | 1 | 3.8 | 1 | 4.3 | 0 | 0.0 | 2 | 5.4 | 3 | 7.3 |
| III | *L. iners* | 33 | 16.5 | 7 | 16.7 | 1 | 7.1 | 6 | 23.1 | 0 | 0.0 | 5 | 29.4 | 10 | 27.0 | 8 | 19.5 |
| IV | Depletion | 39 | 19.5 | 6 | 16.7 | 2 | 14.3 | 4 | 15.4 | 9 | 39.1 | 2 | 11.8 | 7 | 18.9 | 8 | 19.5 |
| V | *L. jensenii* | 3 | 1.5 | 2 | 4.8 | 2 | 14.3 | 1 | 3.8 | 2 | 8.7 | 1 | 5.9 | 0 | 0.0 | 5 | 12.2 |
|  | Total | 200 |  | 42 |  | 14 |  | 26 |  | 23 |  | 17 |  | 37 |  | 41 |  |
|  | *p*-value |  |  |  |  |  | 0.478 |  |  |  | 0.076 |  |  |  | 0.575 |  |  |

CST, community state type.

**Supplementary Table 2.** Analysis of the CST groups (I, II, III, and V) representing the dominant *Lactobacillus* species in the indication for delivery (n=153)

| CST | Dominant species | Total | | PPROM | | PTL | | FTL | | Complication^§^ | |
| --- | --- | --- | --- | --- | --- | --- | --- | --- | --- | --- | --- |
|  |  | n, | (%) | n, | (%) | n, | (%) | n, | (%) | n, | (%) |
| CST I | *L. crispatus* | 81 | 52.6% | 12 | 54.5% | 14 | 50.0% | 44 | 55.0% | 11 | 47.8% |
| CST II | *L. gasseri* | 8 | 5.2% | 1 | 4.5% | 1 | 3.6% | 3 | 3.7% | 3 | 13.0% |
| CST III | *L. iners* | 28 | 18.8% | 4 | 18.2% | 6 | 21.4% | 17 | 21.3% | 1 | 4.3% |
| CST IV | Depletion | 28 | 18.2% | 4 | 18.2% | 6 | 21.4% | 12 | 15.0% | 6 | 26.1% |
| CST V | *L. jensenii* | 8 | 5.2% | 1 | 4.5% | 1 | 3.6% | 4 | 5.0% | 2 | 8.7% |
|  | Total | 153 |  | 22 |  | 28 |  | 80 |  | 23 |  |

^§,^ Pregnant women with medical indication were diagnosed with preterm labor. CST, community state type; PTL, preterm labor; FTL, full-term labor.

Statistical analysis was performed using the χ^2^-test. *p*=0.716

|  | Percentile | Total | Term | | Preterm | | *p*-value |
| --- | --- | --- | --- | --- | --- | --- | --- |
|  |  | *n,* | *n*, | % | *n*, | % |  |
| 14~36 week | > 90% | 100 | 63 | 74.1 | 37 | 50.0 | 0.002 |
|  | ≤ 90% | 59 | 22 | 25.9 | 37 | 50.0 |  |
| > 37 | > 90% | 22 | 22 | 53.7 |  |  |  |
|  | ≤ 90% | 19 | 19 | 46.3 |  |  |  |

**Supplementary Table 3.** Comparison between the frequencies of FTB and PTB according to vaginal *Lactobacillus* abundances in pregnant women (n=200)

FTB, full-term birth; PTB, preterm birth

Statistical analysis was performed by Fisher’s exact test.

**Supplementary Table 4.** Comparison of the frequencies of indications for delivery according to the vaginal *Lactobacillus* abundances

| Relative abundance of *Lactobacillus* | Total | | PPROM | | PTL | | FTL | | Complication^§^ | |
| --- | --- | --- | --- | --- | --- | --- | --- | --- | --- | --- |
|  | n, | (%) | n, | (%) | n, | (%) | n, | (%) | n, | (%) |
| > 90% | 98 | 63.2% | 12 | 46.2% | 18 | 66.7% | 57 | 74.0% | 11 | 44.0% |
| ≤ 90% | 57 | 36.8% | 14 | 53.8% | 9 | 33.3% | 20 | 26.0% | 14 | 56.0% |
|  | 155 |  | 26 |  | 27 |  | 77 |  | 25 |  |

^§,^ Pregnant women with medical indication were diagnosed with preterm labor. PPROM, preterm premature rupture of fetal membranes; PTL, preterm labor; FTL, full-term labor. Statistical analysis was performed using the χ^2^-test. *p*=0.011

**Supplementary Table 5.** Region-specific primers

| Forward primer | 5′-TCGTCGGCAGCGTCAGATGTGTATAAGAGACAGTCGTCGGCAGCGTCAGATGTGTATAAGAGACAGCCTACGGGNGGCWGCAG-3′ |
| --- | --- |
| Reverse primer | 5′-GTCTCGTGGGCTCGGAGATGTGTATAAGAGACAGGTCTCGTGGGCTCGGAGATGTGTATAAGAGACAGGACTACHVGGGTATCTAATCC-3′ |
